# Phthisis and Pneumonia

**Published:** 1900-02-24

**Authors:** 


### Hospital Clinics and Medical Progress.

### PHTHISIS AND PNEUMONIA.

If one were to look upon the word pneumonia as meaning merely inflammation of the lung, there would be no doubt or question that phthisis and pneumonia are intimately related; indeed, one might almost say that pneumonia was the very essence of the phthisical process.

Pneumonia, however, means something else than, something different from inflammation, and if we regard it, as is now generally recognised to be the case, as not being primarily or essentially a disease of the lung, but rather a general fever with a predominating local manifestation in the lung or lungs, the question of the relation between phthisis and pneumonia assumed quite a different aspect. The problem is one which constantly occurs in practice, and it is of great importance that we should be prepared with an authoritative answer to two questions to which it gives rise: first, does the existence of pulmonary tuberculosis render the lung more liable to be attacked by pneumonia? second, does the occurrence of pneumonia determine the establishment of pulmonary tuberculosis? Dr. R. W. Philip,[1] of the Victoria Hospital for Consumption and the Royal Infirmary, Edinburgh, has lately gone carefully into this question, and from what he says it may be taken in regard to the first that the supervention of pneumonia in the course of pulmonary tuberculosis is by no means uncommon, the damaged lung failing more easily a prey to further attack. Not only may the clinical relation be observed frequently, but at the autopsies of persons dying of pneumonia traces of tuberculosis may often be found. A marked example of this was seen in an epidemic of croupous pneumonia which came under observation in connection with a large training institution for boys. A number of the boys died of pneumonia, and on post-mortem examination there were discovered in most cases, in addition to the lesions characteristic of pneumonia, indubitable traces of older tuberculous disease, which in some cases had been latent and in others active.

In some of the cases in which pneumonia occurs as a complication of a pre-existing tuberculosis the course of the pneumonia seems to be undisturbed. In others it runs its course unusually quickly and simply. In regard to this Walshe[2] says: "If lungs already tuberculised become acutely inflamed, convalescence from the pneumonia often takes place as rapidly as if the lungs had previously been sound." Louis says much the same; while Grisolle[3] writes: "These pneumonias do not differ either in respect of cause, or symptoms, or progress from ordinary pneumonia occurring in subjects not predisposed to tuberculous disease."

Still it must be remembered that when tuberculous disease is far advanced, and the breathing space considerably encroached upon, the advent of croupous pneumonia, while comparatively uncommon, is more serious. Another point arising in connection with this side of the subject is, What effect has the advent of pneumonia on the existing pulmonary tuberculosis? Sometimes none. Sometimes, on the other hand, we may note a markedly prejudicial change coming on in the wake of an attack of pneumonia. The more chronic disease may become kindled into activity, and not only may the lung which has been affected by the pneumonia manifest tuberculous disintegration to an alarming degree, but huge excavation may rapidly occur in the other lung. When, then, pneumonia attacks a patient suffering from pulmonary tuberculosis the prognosis must be a guarded one.

The second question is even more important. May pneumonia determine the occurrence of pulmonary tuberculosis? It is but natural to believe that it does. It is, again, only in accordance with what is observed as to the onset of surgical tuberculosis—affections of the joints, &c., after injury—to believe that a lung injured by inflammation should be more liable than when in a healthy condition to become the subject of tuberculosis. There is no doubt that a very large number of practitioners believe that an imperfectly resolved pneumonia frequently passes on to pulmonary tuberculosis, and Niemeyer distinctly lays it down that "every form of pneumonia may, under certain conditions, terminate in cheesy infiltration," adding that "if we say that common acute pneumonia may also lead to cheesy infiltration, we must not be suspected to have made a mistake in the diagnosis of the disease. . . . We sometimes have an opportunity of observing, post-mortem, gradual changes from the red and gray hepatisation, with well marked granulation of the surface of the section, to cheesy infiltration." This view of the matter, however, appears to Dr. Philip to be distinctly erroneous; and in regard to the cases on which it is founded he brings forward the following considerations: (1) In a certain number of instances of apparent delay in the process of resolution in pneumonia, and consequent determination of pulmonary tuberculosis, a faulty interpretation of the sequence of events has resulted from oversight of the fact that the acute pneumonia supervened in an already tuberculous lung. (2) In the limited number of cases where opportunity has been afforded of investigating minutely the lesion left after delayed resolution of croupous pneumonia this lesion has been found to be of a non-tuberculous nature. (3) Occasionally tuberculosis is itself ushered in acutely with symptoms and signs which may be misinterpreted as those of acute croupous pneumonia, and when it is found that the disease continues beyond the usual limit of the latter malady it is assumed erroneously that the case is one of delayed resolution, and then, if later on incontestable evidence of the tubercle bacillus be discovered, it is not difficult to realise how the misconstruction seems justified that croupous pneumonia may suffer delayed resolution and pass into pulmonary tuberculosis. Thus, Dr. Philip concludes that an acute croupous pneumonia is not common as a precedent factor, and is very rarely found in immediate determinant relationship with pulmonary tuberculosis. Which, no doubt, is true; but that pneumonia does sometimes, as is the case with every acute illness by which the strength of a patient is much reduced, become the starting point of a tuberculosis, and that by the local injury it does to the lung tissue it does in some cases become the local determinant cause of the pulmonary tuberculosis seems hardly open to doubt, although probably far less frequently than is often supposed, the sequence of events in the majority of cases falling into one or other of the categories mentioned by Dr. Philip.

[1] Practitioner, Feb. [2] Walshe, Diseases of the Lungs. [3] Grisolle, Traite de la Pneumonie.

